# The Treatment of Infected Wounds

**Published:** 1918-01

**Authors:** 


**The Treatment of Infected Wounds.** By A. Carrel and G. Dehelly. Authorized translation from the French by Herbert Child, M. D., formerly Surgeon French Red Cross, Capt. R. A. M. C. (Ty.), with an introduction by Sir Anthony A. Bowlby, F.R.C.S., Temporary Surgeon General, Army Medical Service. 12mo, cloth, 250 pages, 97 illustrations. $2.00. New York, Paul B. Hoeber, 1917.

---

This monograph presents one complete method of treatment and gives a concise history of its development. "The method is based upon the employment, rigorously controlled by the microscope, of an approved agent, under conditions of contact, of concentration, and of duration, established by direct experiment upon infected wounds." The agent is Dakin's solution of hypochloride of soda, which acts by chemiotherapy; but first the wound undergoes a thorough "mechanical cleansing" or surgical preparation for treatment by the solution. The technic of all these procedures is carefully described. The preparation and application of the solution, the routine daily examination of wounds—clinical and bacteriological—to note the progress of sterilization, the keeping of records, and the final closure of sterile wounds, are all set forth with sufficient detail to enable any surgeon to use the method understandingly. Forty pages, well illustrated, discuss the results of this treatment, including statistics of successes and failures. Very little space is devoted to the controversies that have followed the introduction of this method, but surgeons are urged to use the method "in its entirety" before deciding pro or con. A very complete index facilitates reference to any topic treated by the authors.—F. W. B.

